# Reconstruction of Transcription Control Networks in Mollicutes by High-Throughput Identification of Promoters

**DOI:** 10.3389/fmicb.2016.01977

**Published:** 2016-12-06

**Authors:** Gleb Y. Fisunov, Irina A. Garanina, Daria V. Evsyutina, Tatiana A. Semashko, Anastasia S. Nikitina, Vadim M. Govorun

**Affiliations:** ^1^Federal Research and Clinical Centre of Physical-Chemical MedicineMoscow, Russia; ^2^Shemyakin–Ovchinnikov Institute of Bioorganic Chemistry, the Russian Academy of SciencesMoscow, Russia; ^3^Moscow Institute of Physics and TechnologyMoscow, Russia

**Keywords:** Mollicutes, mycoplasma, spiroplasma, acholeplasma, transcriptomics, promoter, transcription factors

## Abstract

Bacteria of the class Mollicutes have significantly reduced genomes and gene expression control systems. They are also efficient pathogens that can colonize a broad range of hosts including plants and animals. Despite their simplicity, Mollicutes demonstrate complex transcriptional responses to various conditions, which contradicts their reduction in gene expression regulation mechanisms. We analyzed the conservation and distribution of transcription regulators across the 50 Mollicutes species. The majority of the transcription factors regulate transport and metabolism, and there are four transcription factors that demonstrate significant conservation across the analyzed bacteria. These factors include repressors of chaperone HrcA, cell cycle regulator MraZ and two regulators with unclear function from the WhiA and YebC/PmpR families. We then used three representative species of the major clades of Mollicutes (*Acholeplasma laidlawii*, *Spiroplasma melliferum*, and *Mycoplasma gallisepticum*) to perform promoter mapping and activity quantitation. We revealed that Mollicutes evolved towards a promoter architecture simplification that correlates with a diminishing role of transcription regulation and an increase in transcriptional noise. Using the identified operons structure and a comparative genomics approach, we reconstructed the transcription control networks for these three species. The organization of the networks reflects the adaptation of bacteria to specific conditions and hosts.

## Introduction

Class Mollicutes are a specialized clade of Gram-positive bacteria that lack a cell wall and have significant genome reduction. The genome size of Mollicutes ranges from approximately 0.5 Mb in Bermuda grass white leaf phytoplasma or *Mycoplasma genitalium* to nearly 2 Mb in *Acholeplasma brassicae*. Most known Mollicutes are parasites, and some are saprophytes. Despite their reduced genome, Mollicutes demonstrate high adaptive potential within a parasitic niche. The range of their hosts is wide and includes plants (Gasparich, 2010), arthropods (Lo et al., 2013), and vertebrates (Rosengarten et al., 2000).

Genome reduction acts as a force that directs the evolution of the transcriptional regulatory network. A comparison of free-living organisms and phylogenetically related symbionts revealed general patterns of the reorganization of that network (Galán-Vásquez et al., 2016). During the process of genome reduction, bacteria first lose regulators that regulate the single operon, primarily genes involved in the response to environmental stimuli. Thus, only the regulators of the vital cellular processes are retained in the reduced genomes. Parasitic Mollicutes, such as Mycoplasma and Spiroplasma, live attached to or inside the host eukaryotic cells in a relatively stable environment, whereas their close relatives, *Acholeplasma* sp., which are characterized as commensals and saprophytes, are exposed to a broad range of environmental perturbations. The difference in living conditions can dramatically affect the transcriptional regulatory networks of these species. Mollicutes were previously not considered in wide comparative genomics studies aimed at the reconstruction of transcriptional regulatory systems (Madan Babu et al., 2006; van Hijum et al., 2009; Faria et al., 2014), nor were they included in commonly used databases of transcriptional regulators such as RegPrecise (Novichkov et al., 2013) or CollecTF. Attempts to elucidate the transcriptional regulation in Mollicutes using high-throughput technologies (Lozada-Chávez et al., 2006; Güell et al., 2009; Mazin et al., 2014) resulted in limited progress. Regulation via an alternative sigma factor was demonstrated for *M. genitalium* (Torres-Puig et al., 2015). The structure of the core promoter also modulates transcriptional response to stress (Mazin et al., 2014). Our previous study explained how the regulation of thousands of genes in *M. gallisepticum* could be achieved without specific regulators. However, the function of potential transcription factors in genomes of reduced bacteria, such as *M. gallisepticum*, remains unknown. A response to stress without transcription factors calls their function into question.

Mollicutes are interesting subjects in the context of the minimal cell problem. The problem is the minimal repertoire of genes that support a self-replicating cell growing in a medium that contains a variety of nutrients and cofactors. The development of technologies of synthesis and transplantation of the artificial genome makes it possible to experimentally prove the concept of the minimal cell (Hutchison et al., 2016). Mollicutes show a significant reduction in metabolic pathways, which primarily concentrate around the generation of energy (Fisunov et al., 2011). Most Mollicutes import all nutrients and cofactors due to a complete loss of respective synthetic pathways (Vanyushkina et al., 2014). Recent progress of bacterial genomics indicate that a minimal set of genes can instead be viewed as a minimal set of functions in which the same function may be carried out by different sets of genes. From the perspective of gene expression regulation, there is a problem by which functions require regulation on the level of transcription, if any. Mollicutes may serve as a model to study a minimal set of regulators. There is a significant difference between minimal cells and Mollicutes. The latter developed a broad repertoire of proteins involved in host-pathogen interactions and the import of nutrients due to their parasitic lifestyle. In this context, even a reduced repertoire of regulators may be solely involved in the regulation of genes involved in the interactions with the host.

Precise transcription start site (TSS) mapping at a single-nucleotide resolution may significantly contribute to understanding gene expression organization and control. However, there are few data on TSS mapping across the bacteria despite numerous transcriptomics projects (Sharma et al., 2010; McClure et al., 2013; Wade, 2015). Recent advances in bacterial transcriptomics demonstrate a complex organization of the transcriptional apparatus (Nicolas et al., 2012; Junier et al., 2016). Genes and operons may have complex systems of transcription initiation including multiple promoters and TSSs (Maxson and Darwin, 2006; Rosario and Tan, 2016). Thus, the genome-scale identification of TSSs represents another layer of genome information that cannot be derived solely from its sequence but is crucial for its function.

In the current work, we carried out whole-genome mapping of transcription start sites (TSSs) of *Acholeplasma laidlawii* and *Spiroplasma melliferum* and used our previous data on *Mycoplasma gallisepticum* (Mazin et al., 2014) for a comparative study of promoters and transcription unit organization and regulation across Mollicutes. In this set, *A. laidlawii* represents a clade of Mollicutes that underwent less reduction (1.5 Mb genome), whereas *S. melliferum* and *M. gallisepticum* are average Mollicutes with a genome size of approximately 1 Mb. *A. laidlawii* is likely a saprophyte or a facultative plant pathogen. *S. melliferum* has two hosts (angiosperm plants and honeybees) and is predominantly pathogenic in the latter. *M. gallisepticum* is a bird pathogen closely related to the human pathogens *M. pneumoniae* and *M. genitalium*. Mapping TSSs enabled us to study the promoters’ structure and to identify potential transcription factor binding sites and regulons in respective species.

## Materials and Methods

### Cell Culturing

*Mycoplasma gallisepticum S6* was cultivated in liquid medium containing tryptose 20 g/L, Tris 3 g/L, NaCl 5 g/L, KCl 5 g/L, yeast dialysate (5%), horse serum (10%), and glucose 1% at pH 7.4 and 37°C in aerobic conditions and exposed to heat stress conditions (46°C for 15 min) as described previously (Gorbachev et al., 2013). For the experiment, cells were cultivated until stationary phase (approximately 20 h), and the next passage was performed in 1:10 dilution. The cells were then cultivated 12 h for harvesting (mid-exponential phase).

*Acholeplasma laidlawii PG-8A* was cultivated in modified Edward’s medium (liquid): tryptose 20 g/L, NaCl 5 g/L, NaOAc 5 g/L, KCl 1.3 g/L, Tris 3 g/L, yeast dialysate 5%, horse serum 6%, glucose 0.5%, and pH 7.6 at 37°C in aerobic conditions (Fisunov et al., 2011). For the experiment, cells were cultivated until stationary phase (approximately 20 h), and the next passage was performed in 1:100 dilution. The cells were then cultivated for 16 h for harvesting (mid-exponential phase). Heat stress was performed at 44°C for 15 min.

*Spiroplasma melliferum KC3* was cultivated in SP4 medium (liquid): tryptone 10 g/L, peptone 10 g/L, sorbitol 70 g/L, yeast extract 7 g/L, brain-heart infusion 2.5 g/L, NaCl 4.5 g/L, sucrose 1%, fructose 1%, glucose 0.8%, horse serum 10%, and pH 7.6 at 30°C in aerobic conditions (Vanyushkina et al., 2014). For the experiment, cells were cultivated until stationary phase (approximately 24 h), and the next passage was performed in 1:10 dilution. The cells were then cultivated for 10 h for harvesting (mid-exponential phase). Heat stress was performed at 37°C for 15 min.

### RNA Extraction and Preparation of 5′-ERS Libraries

Preparation of 5′-ERS (5′-end Enriched RNA Sequencing) libraries was performed as previously described (Mazin et al., 2014). Cells from 1 ml of culture were centrifuged for 10 min at 8,000 *g*, resuspended in wash buffer (100 mM Tris, 100 mM NaCl, 2 mM MgCl_2_, pH 7.4) and lysed in TRIzol LS reagent (Life Technologies) at a 1:3 ratio of resuspended cells:TRIzol LS (v/v). The lysates were extracted with chloroform, and the aqueous phase was purified with a PureLink RNA Mini Kit (Ambion) to remove tRNA or used directly to precipitate RNA with the addition of an equal volume of isopropanol.

Approximately 20 μg of total RNA was fragmented into 200 bp via chemical fragmentation (100 mM ZnSO_4_, 100 mM Tris, pH = 7.0 at 70°C for 15 min). The fragmentation reaction was stopped with 20 mM EDTA (pH = 8.0). The fragmented RNA was end-repaired with T4 polynucleotide kinase according to the manufacturer’s protocol (Thermo).

Fragmented end-repaired RNA was treated with Terminator exonuclease (Epicentre). This process resulted in the degradation of the non-primary 5′-end RNA fragments, whereas the primary 5′-fragments were protected by the tri-phosphate groups on their 5′-ends. As chemical fragmentation leaves phosphates randomly on fragment ends, an end-repair procedure was used to enhance the degradation of non-primary 5′-end fragments (e.g., with 5′-OH), which otherwise undergo adapter ligation and cDNA synthesis (if they have 3′-OH) and to rescue primary 5′-end fragments with 3′-phosphate (via 3′-phosphatase activity of T4 PNK). The addition of an end-repair procedure increases signal (e.g., coverage of primary 5′-ends) and decreases background. Then, the RNA was precipitated with isopropanol and treated with tobacco acid phosphatase (Epicentre) to remove the pyrophosphate groups. Next, the RNA was precipitated via isopropanol and used for strand-specific ds-cDNA preparation according to the standard protocol for SOLiD libraries. The sample cDNA was normalized in one round as described above and used to prepare SOLiD libraries according to the standard protocol. The quality of the RNA, fragmented RNA and cDNA libraries was assayed with an Agilent 2100 Bioanalyzer system (Agilent).

### Transcription Factors Conservation Analysis

Ortholog clusters for 50 Mollicute species were identified using protein BLAST. To exclude paralogs, we calculated thresholds to sequence identity for all pairs of the analyzed genomes. We scanned proteomes of all species against a fully conserved domain database (Marchler-Bauer et al., 2015) and extracted proteins with a helix-turn-helix motif. False-positive matches with DNA-binding proteins unrelated to the regulation of transcription were manually removed.

### Reconstruction of Mollicutes Phylogeny

From ortholog clusters, we extracted proteins conserved between all analyzed species. All clusters were aligned with MUSCLE (Edgar, 2004) and then concatenated together. We performed phylogenetic analysis using a Neighbor-joining algorithm with 1,000 bootstraps implemented in the PHYLIP package.

### TSS Identification

All reads with average quality values below 15 were discarded. The reads were truncated at 3′-end and first 25 bases were used for mapping. The reads were mapped to the *M. gallisepticum* strain *S6* genome (CP006916.3 assembly) using the Bowtie software (Langmead et al., 2009) with the following parameters: bowtie –trim3 23 -f -C -v 3 -y -a –best –strata -S. Each match for the reads that was mapped to multiple positions was treated as an independent read. The results were nearly the same when only the uniquely mapped reads were used.

To identify TSSs, we searched for a local maximum in the read coverage that was supported by at least five reads. We then modeled the coverage at each local maximum while considering 5 nt in each direction as background using a GLM (Generalized Linear Model) with a quasi-binomial distribution and controlling the overdispersion parameter to be no lower than 1. We used a quasi-log likelihood test to identify significant coverage peaks (BH-corrected *p*-value < 0.05). The strength of the promoters was calculated as the number of reads covered by TSS normalized to library size. We manually verified all TSSs, removed false-positives within coding regions or appended those missed.

### Data Access

Transcriptomics data were uploaded to the NCBI SRA database under project ids PRJNA325091, PRJNA325092, and PRJNA325094.

## Results

### Transcription Factors Distribution Across the Mollicutes

**FIGURE 1:**
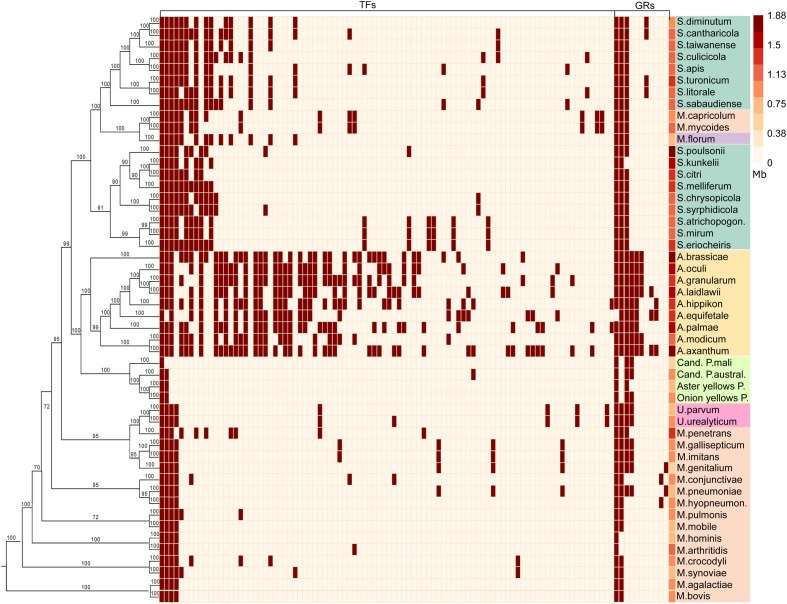
**Regulators conservation in Mollicutes: distribution of transcription factors (TFs) and global regulators (GRs); the last column represents the genome size.** Plot shows the presence (brown) or absence (white) of regulators conserved in at least two analyzed genomes. Phylogenetic tree was constructed based on alignments of orthologs conserved between all species. Background colors for species: yellow - *Acholeplasma* sp., aquamarine - *Spiroplasma* sp., orange - *Mycoplasma* sp., lime - *Phytoplasma* sp., violet - *Mesoplasma florum*, pink - *Ureaplasma*.

**FIGURE 2:**
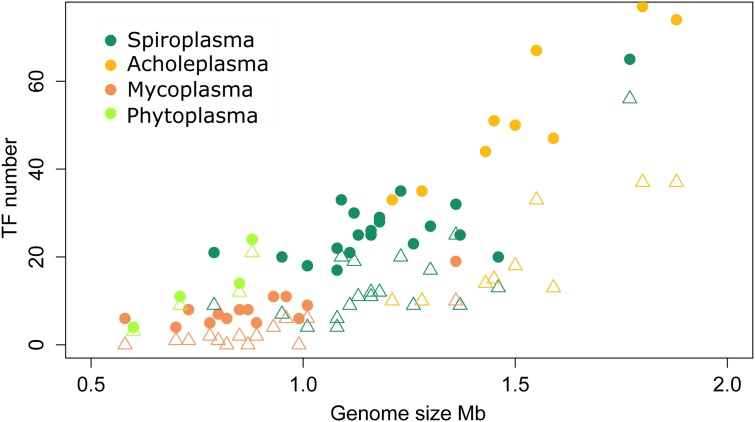
**Transcription factors number variation across the Mollicutes.** Circles represent the total number of TFs; triangles show the number of non-conserved TFs only present in one of the analyzed genomes.

We studied the distribution and conservation of transcriptional regulators in a representative group of 50 species of Mollicutes (**Figure 1**). To identify transcription factors (TFs), we comprehensively searched for the genes with high homology with known TFs or proteins with a helix-turn-helix DNA-binding domain. The general genome reduction in Mollicutes involves a massive loss of transcriptional regulators. In *Escherichia coli* and *Bacillus subtilis*, core cellular functions, including genome replication and protein translation, require nearly 5 and 6% of the proteome, respectively. In Mollicutes, this fraction progressively increases with genome reduction: 12.6% in *A. laidlawii*, 14% in *S. melliferum*, and 18.7% in *M. gallisepticum*, which indicates a progressive loss of dispensable functions (**Table 1**). The average genomic repertoire of TFs in bacteria is 131 proteins per species. For example, 290 transcription factors were described for *E. coli* and 238 for *B. subtilis* (Charoensawan et al., 2010). The average number of transcriptional regulators in the genomes of Mollicutes is approximately 25, which represents approximately 2.5% of all genes. The fraction of TFs decreased with the genome reduction, reaching a maximum of 4.5% in Acholeplasma brassicae and a minimum in *Mycoplasma hominis* (0.07%). In general, the number of TFs demonstrates a nearly linear decrease in the reduction of the genome size until approximately 1 Mb (the *S. melliferum* genome size was estimated from the contigs’ length). The number of TFs plateaus at 5-10 TFs per genome (**Figure 2**). The studied set of species retains a significant number of non-conserved regulators, which also decreases with the genome reduction.

**Table 1:** The number of replication, translation, and transcription regulation proteins in bacteria.

	Replication (GO:0006260)		Transcription (number of TFs)		Translation (GO:0006412)		Total proteins
*Escherchia coli*	67	1.4%	290	6.0%	158	3.3%	4820
*Bacillus subtillis*	65	1.5%	238	5.6%	183	4.3%	4243
*Acholeplasma laidlawii*	30	2.2%	52	3.8%	143	10.4%	1381
*Spiroplasma melliferum*	24	2.1%	23	2%	137	11.9%	1154
*Mycoplasma gallisepticum*	20	2.4%	10	1.2%	136	16.3%	835


The pan-genome of the studied Mollicutes encompasses 92 conserved TFs, most of which are distributed within acholeplasmas. Mollicutes are divided into three groups on the basis of the TFs number, conservation and the genome size. These groups form distinct clusters on both the TFs conservation plot (**Figure 1**) and the distribution of genome sizes and number of TFs (**Figure 2**). The first represents acholeplasmas and shows a relatively high number of conserved TFs. The second group includes spiroplasmas with an average TFs number of 27. The two latter groups feature group-specific TFs, which are conserved in one and absent in another. The third group consists of mycoplasmas, ureaplasmas, and phytoplasmas. This group demonstrates a high number of TF mosaicism and an extremely low number of TFs conserved within the group and with other Mollicutes. There are only four conserved TFs within more than one group of Mollicutes: the repressor of chaperones of HrcA, which are poorly studied TFs from the WhiA and YebC/PmpR families, as well as MraZ, a regulator of the cell cycle. However, the latter is lost in several species including most acholeplasmas. We propose that in these cases, the functions of MraZ were taken by a non-orthologous protein. These TFs are of particular interest because two of them (HrcA and MraZ) control core cellular function. The two others most likely control important but as yet unknown functions. The binding sites of HrcA (Chang et al., 2008) and MraZ (Fisunov et al., 2016) in Mollicutes are known and are highly conserved. The binding site of the YebC/PmpR-family TF was found in *A. laidlawii* and controls inorganic pyrophosphatase and genes with unknown function. Its binding site in other species is unclear.

The binding site of the WhiA-family TF was predicted using two approaches. The first, as described earlier, resulted in the identification of putative binding sites in *S. melliferum* and *M. gallisepticum* (see below). However, the functions of these TFs were substantially different according to the prediction. Taking into account the ubiquitous distribution of WhiA homologs, we propose that this prediction may be incorrect and that WhiA homologs control more core cellular functions. Alternatively, Jakimowicz et al. (2006) demonstrated that WhiA may control replication genes in *Streptomyces*. Hence, we searched for common conserved motifs in the promoters of the respective genes in Mollicutes. We identified common motifs for acholeplasmas and spiroplasmas, which were similar in both clades (Supplementary Material S1). We did not identify conserved motifs common for mycoplasmas in either a reference-independent search or by searching for the identified acholeplasma-spiroplasma motif.

We also analyzed the distribution and conservation of global regulators (GRs) (**Figure 1**). For GRs, we denoted proteins that regulate a broad spectrum of genes through the modulation of RNA-polymerase activity. We included in this group both alternative sigma factors and other proteins that alter promoter recognition. We identified a set of alternative sigma factors in the Mollicutes genomes and SpxA and SpoT (ppGpp, guanosine-tetraphosphate synthase) proteins, which regulate transcription through different mechanisms. SpxA binds to RNA-polymerase alpha subunits and interacts with the region upstream of the core promoter. SpxA is involved in the host-pathogen interaction and controls oxidative stress response in bacteria (Rochat et al., 2012; Kajfasz et al., 2015). The primary role of ppGpp in the transcriptional regulation in Gram-positive bacteria appears to be a decrease in the GTP pool, which leads to a decrease in the transcription initiation rate on promoters with a G initiator nucleotide (Krásný and Gourse, 2004). Mollicutes have one highly conserved sigma factor, a homolog of the main sigma-70 factor, and a set of putative alternative sigma factors with promoter binding domains. These proteins are primarily species-specific, are partial homologs of sigma-70 factor and frequently have only -35 box-binding domain. Contrary to other Mollicutes, phytoplasmas have multiple homologs of sigma factors in highly reduced genomes. We propose that these proteins took on the function of transcription factors because phytoplasmas have extremely low number of conserved TFs compared with equally reduced mycoplasmas. We included Hpr (phosphocarrier protein of the sugar phosphotransferase system) kinase in the list of global regulators as well because it may act with a specific TF in a process of global regulation known as carbon catabolite repression (Deutscher, 2008). Hpr kinase is conserved in nearly all Mollicutes with the exception of phytoplasmas. However, there appears to be no such universal TF associated with HprK in all Mollicutes.

### Mollicutes Evolved Toward Promoter Simplification and An Increase in the Transcriptional Noise

**FIGURE 3:**
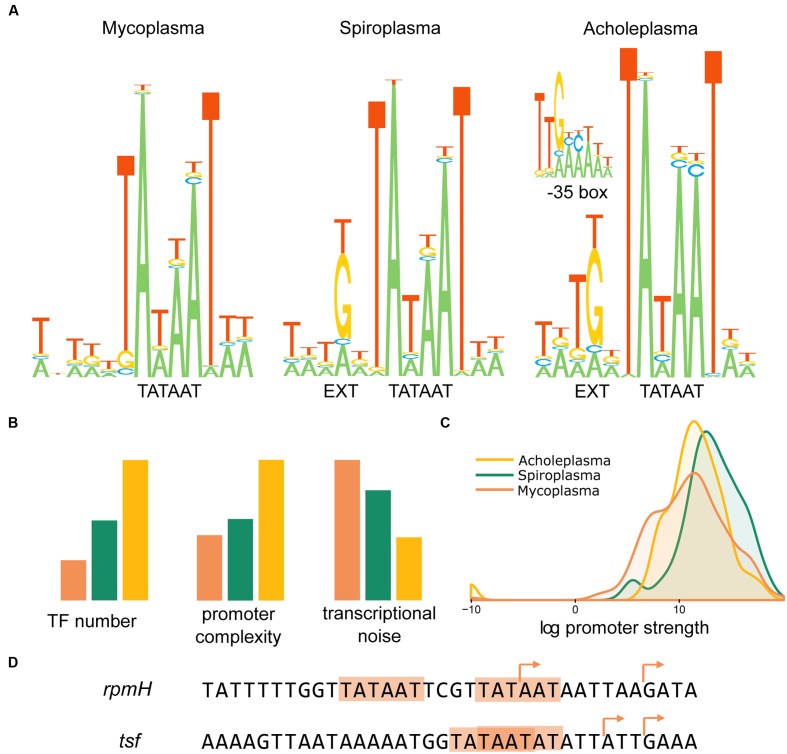
**Promoter structure of *Acholeplasma laidlawii*, *Spiroplasma melliferum*, and *Mycoplasma gallisepticum*.** **(A)** Core-promoter structure in three species. **(B)** The complexity of transcriptional control in three species. Promoter complexity was measured as the number of nucleotides with high informational content. Transcriptional noise was measured as the relative number of intragenic and antisense promoters. **(C)** The dynamic range of the promoters’ strength in three species. The strength of the promoter was measured as the normalized number of reads covering TSS. **(D)** Examples of tandem promoters in *M. gallisepticum*. Arrows indicate experimentally validated TSSs.

Algorithmic TSSs identification with subsequent manual curation resulted in 607 TSSs for *A. laidlawii* (**Supplementary Table S1**), 240 for *S. melliferum* (**Supplementary Table S2**) and 430 for *M. gallisepticum* (**Supplementary Table S3**). The core promoter structure is similar in all species (**Figure 3A**). The most conserved elements are the -10 box (TAWAAT), EXT element (TRTG) and initiator nucleotide (G or A). The most frequent spacer between the TSS and -10 box is 6 nt; however, 5 and 7 nt spacers also exist. One of the determinants for a spacer that is other than 6 nt in length is the non-optimal initiator nucleotide (C or T) at an optimal TSS position. In *A. laidlawii* and *S. melliferum*, there is also a pronounced -35 box (TTGACA), whereas in *M. gallisepticum* it is rare and less conserved. In general, *A. laidlawii* demonstrates the most pronounced promoter structure, regardless of whether *M. gallisepticum* shows promoter degeneration.

Bacteria demonstrate a significant level of transcriptional noise arising at spurious promoters (Lloréns-Rico et al., 2016). The number of sRNAs in the genome exponentially depends on the genome AT content. Our study found that the structure of the promoter could also affect the sRNA expression. The fraction of intragenic and antisense TSSs in the genomes of the three analyzed Mollicutes varies considerably despite a similar AT content (**Figure 3B**). Transcriptional noise correlates with promoter complexity and is calculated as the number of nucleotides with high informational content (**Figure 3B**). We assume that promoters simplified in Mollicutes along with the genome reduction and the loss of transcriptional regulators. For instance, we observed an extremely high number of spurious promoters within cassettes of *vlhA* hemagglutinins of *M. gallisepticum*. Approximately 40% of TSSs in *vlhA* cassettes emerge from the promoters of this type. The expansion of antisense transcription could compensate for TF deficiency and improve adaptive capabilities in Mycoplasmas.

The 5′-ERS (5′-end Enriched RNA Sequencing) method enables not only the identification of TSSs positions but also the ability to measure the activity of the respective promoters. The difference in the degree of promoter reduction between more and less reduced species may result in an alteration of the dynamic range of the promoters’ activities. However, all three species demonstrated comparable dynamic ranges of promoter strength (**Figure 3C**). A relative reduction in mycoplasma promoters does not change the dynamic range of the promoters’ strength of protein coding genes on a genome scale. Notably, *M. gallisepticum* shows a relatively increased number of weak promoters. In addition to overall promoter simplification, *M. gallisepticum* demonstrates a phenomenon of tandem promoters. These promoters consist of two -10 boxes located near each other with a short spacer or even overlapping. These promoters have two TSSs, respectively, for example, promoters of *rpmH and tsf* genes (**Figure 3D**). Tandem promoters likely evolved as an alternative to optimal promoters with pronounced Ext and -35 elements.

### *De Novo* Prediction of TFs Binding Sites and Targets Using a Comparative Genomics Approach Coupled with Precise TSS Identification

We used experimentally identified positions of TSSs (**Supplementary Tables S1**–**S3**) to determine the regulatory potential of the studied species. We were primarily focused on transcription factors; there are also homologs of global transcriptional regulators in Mollicutes. Transcription factors (TFs) can be divided into two groups: TFs located within the operon and isolated TFs. The operon structures were inferred from the TSS map. We then studied the conservation of the operon structure. In this approach, we assumed that the conservation of a TF within the operon is due to a functional link between them rather than due to a random event. For isolated TFs, we performed essentially the same procedure with the reference to the potential target operon’s structure. We assumed that there is a chance that isolated TFs belong to their own regulon in at least some species. To predict TF binding sites, we used sequences of closely related bacteria. We searched for homologs of transcription factors with no less than 60% identity between protein sequences in at least three bacteria. We presumed that regulators within operons are regulated by TFs within this operon because autoregulation is a very frequent phenomenon across bacteria (Martínez-Antonio and Collado-Vides, 2003). We identified conservative fragments accepted as candidates to the TF binding site via aligning sequences of the upstream and downstream TSSs of homologous TFs. Mollicutes have no well-studied closely related species with similar GC contents; we thus had few indicators to ensure binding site prediction. They are as follows:

(1) High informational content (Wunderlich and Mirny, 2009) in combination with increased GC content: analyzed species have a low GC content (approximately 30%); however, functionally significant regions have a higher GC content compared with intergenic regions.
(2) Presence of palindromes or repetitive sequences (Rodionov, 2007).
(3) Similar words with known motifs: we used a manually curated database of regulons and transcription factors (RegPrecise) (Novichkov et al., 2013). For example, all predicted sites of the TetR transcription factor family have a TGA motif present in the binding sites of Bacillales.

Based on the predicted motifs, we constructed Positional Weighted Matrices and scanned genomes to predict the putative targets of the regulators.

### Acholeplasmas have the Most Complex Transcriptional Regulation Across Mollicutes

In general, Acholeplasmas have a broad range of transcriptional regulators compared with other clades of Mollicutes. We constructed a transcriptional regulatory network as an example for that of *A. laidlawii.* There are 53 conserved TFs in *A. laidlawii*; 39 of these are located within operons including the only TF with a known binding site: HrcA. There is also one case of the fusion of a TF with an enzyme (ACL_RS02790). The search for potential regulatory sequences using the simultaneous conservation of TF, operon and promoter resulted in the identification of 28 potential binding sites (**Supplementary Table S4**). The rest demonstrated relatively low homology with known proteins. Most of the identified putative regulatory sequences showed a regular structure, e.g., inverted or direct repeats, which is in accordance with the organization of known binding sites in bacteria. Only five were irregular. The regulons of 21 TFs were limited by their own operon. The conservation approach also revealed several potential binding sites of isolated TFs. As we expected, their regulons encompassed multiple operons. We identified potential binding sites for 6 isolated TFs. In total, our approach enabled the identification of 34 of the 52 potential binding sites. For the rest, we were not able to identify conserved motifs most likely due to the low conservation of the respective TFs. An analysis of TF conservation showed that most are conserved among acholeplasmas; however, four have close homologs outside Mollicutes.

**FIGURE 4:**
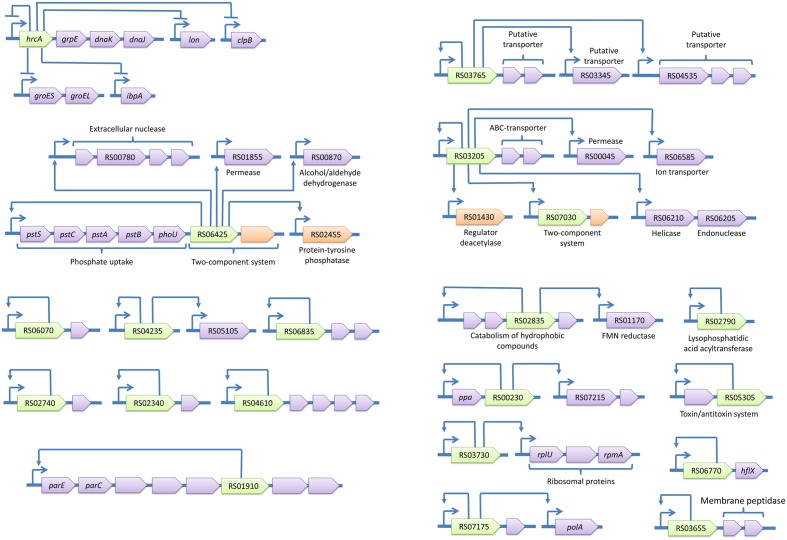
**Transcriptional regulons of *A. laidlawii.*** Green genes represent TFs with predicted binding sites, violet genes are target genes and orange genes are non-transcriptional regulators. TSSs of operons are shown by arrows upstream of the genes; other arrows show predicted regulatory relationships between genes.

**FIGURE 5:**
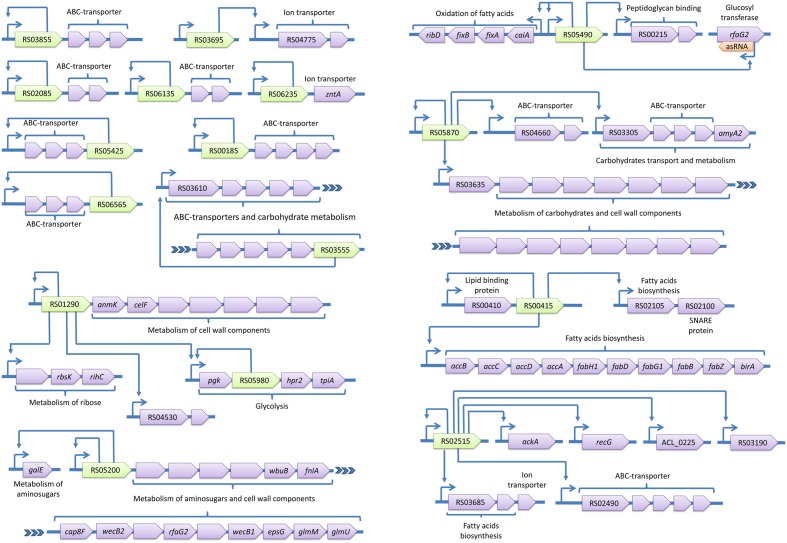
**Transcriptional regulons of *A. laidlawii* (continued).** Green genes represent TFs with predicted binding sites, violet genes are target genes and orange genes are non-transcriptional regulators. TSSs of operons are shown by arrows upstream of the genes; other arrows show predicted regulatory relationships between genes.

The identified regulons are summarized in **Figures 4** and **5**. The spectrum of functions controlled by TFs in *A. laidlawii* includes membrane transport and metabolism, the modulation of translation, protein folding, DNA supercoiling and a toxin-antitoxin system. Twenty TFs control operons involved in membrane transport and metabolism. Seven of these TFs control solely membrane transporters. One TF is likely involved in the modulation of translation. Only HrcA controls stress-related functions (protein folding). The rest of the TFs control regulons with an unclear function. Some of their targets appear to be membrane proteins; they may thus be involved in nutrient uptake.

The most represented group of TF-controlled metabolic pathways is the metabolism of membrane components and carbohydrates and the catabolism of cell wall components (8 TFs). Enzymes of these groups are often combined within one regulon. For example, ACL_RS01290 controls enzymes related to the glycolysis and metabolism of murein and ribose. *laidlawii* Two regulatory systems appear to form cascades (ACL_RS03205 - ACL_RS07030 and ACL_RS01290 - ACL_RS05980).

*Acholeplasma laidlawii* demonstrates horizontal gene transfer of TFs (Supplementary Material S1). Two TFs were transferred with respective operons; however, one ACL_RS05490 controls multiple operons and resides outside them. The spectrum of TF-donor bacteria includes Carnobacterium, Blautia, Eubacterium, Lachnospiraceae, and Olsenella. Isolated and low-conserved TFs in Mollicutes pose a theoretical problem. If they were recently acquired from yet unknown species, they are useless without respective target genes. These TFs may represent an evolutionary source for the subsequent development of new regulatory pathways and adaptation to new environmental conditions (Lercher and Pál, 2008; Wiedenbeck and Cohan, 2011).

### Transcriptional Control Network in *S. melliferum* Reflects an Adaptation to the Switch between Two Hosts

**FIGURE 6:**
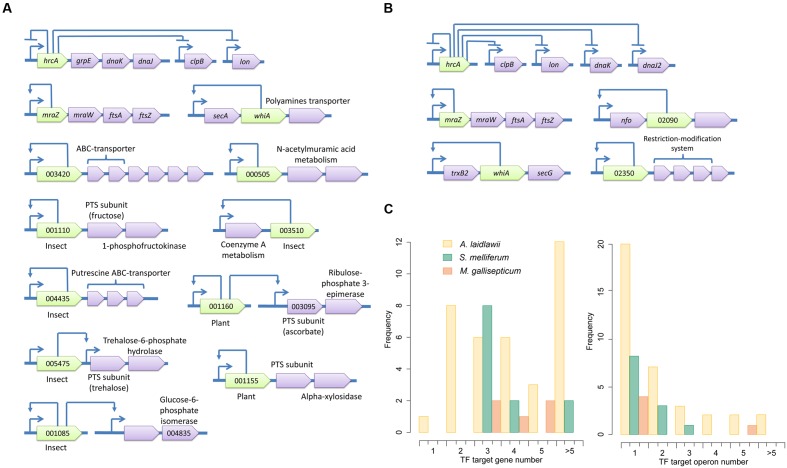
**Transcriptional regulons of *S. melliferum***(A)**** and *M. gallisepticum* **(B)**. Green genes represent TFs with predicted binding sites, violet genes are target genes and orange genes are non-transcriptional regulators. TSSs of operons are shown by arrows upstream of the genes; other arrows show predicted regulatory relationships between genes. **(C)** The summary of TF-controlled genes and operon numbers in three species.

Spiroplasmas occupy an intermediate position between TF-rich acholeplasmas and reduced mycoplasmas. *S. melliferum* has 23 conserved TFs; 14 of these reside within operons. We identified putative binding sites for 12 TFs (**Supplementary Table S5**), 11 of which are located within operons (**Figure 6A**). One of the problems with the identification of putative binding sites in *S. melliferum* is the relatively high number of isolated TFs.

The functional variety of TF-controlled genes includes cell division, membrane transport and metabolism. The majority of TFs (10) with identified binding sites control metabolic functions. In particular, they control the uptake and metabolism of carbohydrates. Other TF-controlled genes code for proteins involved in pantothenate/coenzyme A metabolism, polyamines transport, and protein export. We note that the putative regulator of pantothenate/coenzyme A metabolism is a member of an ambiguous protein family that encompasses both enzymes and transcriptional repressors (ROK-family IPR000600). Thus, this regulator may not be a TF but rather an enzyme of the CoA biosynthetic pathway. *S. melliferum* lives in two hosts and feeds on substances with very different nutritional compositions: insect hemolymph and phloem sap of vascular plants. Insect hemolymph is rich in monosaccharides and trehalose (Wyatt, 1961) and contains substantial amount of putrescine (Birnbaum et al., 1988), whereas phloem sap is rich in sucrose (Hayashi and Chino, 1990), contains significant amount of ascorbate (Hancock et al., 2003), does not contain either glucose or fructose (Hayashi and Chino, 1990) and is depleted of group B vitamins (Salem et al., 2014). We identified seven TFs with known putative binding sites involved in the regulation of nutrient uptake during the host change. TFs responsible for the regulation of transport and metabolism of glucose (SPM_001085), fructose (SPM_001110), trehalose (SPM_005475), putrescine (SPM_004435), and pantothenate and/or coenzyme A metabolism (SPM_003510) are insect-specific, whereas TFs regulating ascorbate (SPM_001160) and xylose (SPM_001155) uptake and metabolism are plant-specific. Generally, parasitic Mollicutes lack all metabolic pathways of vitamins and cofactors biosynthesis because they are usually available from hosts. However, some pathways, such as the coenzyme A biosynthesis pathway from pantothenate, are retained in *S. melliferum* and are controlled by the specific transcription factor. Phloem sap does not contain pantothenate, and coenzyme A is directly imported to Spiroplasma during plant infection. During persistence within the insect host, *S. melliferum* can uptake pantothenate from hemolymph to subsequently synthesize coenzyme A [27].

*Spiroplasma melliferum* demonstrates a gradual loss of HrcA binding sites. There are 3 HrcA-controlled promoters of the *dnaK* operon, *clpB*, and *lon*. Promoters of the *dnaK* operon and *clpB* have two copies of CIRCE. However, both copies in the *dnaK* promoter have four mutations and one copy in *clpB* promoter has three mutations; in each case, this disrupts one of the repeats. Comparison with *M. gallisepticum* shows that functional CIRCE have no more than two mutations, which leaves one in the *clpB* promoter and one in the *lon* promoter.

Identified putative TF binding sites of *S. melliferum* and *M. gallisepticum* have interesting commonalities. They avoid using G and C nucleotides on the same strand (in the same repeat). However, the phenomenon of compositional bias is widespread in bacteria, especially among pathogenes and endosymbionts (Saha et al., 2014). In the case of a palindrome, one of the repeats contains all G’s and other all C’s on a given strand. The sole exception is the HrcA binding site, which is extremely conserved in Bacteria and evolved outside of Mollicutes. *A. laidlawii* does not demonstrate this phenomenon. We hypothesize that the nucleotide composition bias of TF binding sites may be caused by a selection toward becoming more resistant to cytosine deamination.

### Mycoplasmas Demonstrate the Most Simplified Transcriptional Regulation

*Mycoplasma gallisepticum* shows the smallest repertoire of transcription factors among the studied species. However, this situation is typical for mycoplasmas. There are 10 potential TFs in *M. gallisepticum* (**Supplementary Table S6**). The binding sites of HrcA and MraZ are known. Other TF are significantly less conserved. Close homologs were found only in *M. imitans* (and once solely in *M. hominis*), which significantly reduces the reliability of the conservation approach because many intergenic regions in *M. imitans* show extreme homology with *M. gallisepticum*. Eight potential TFs of *M. gallisepticum* reside within operons. Regularly conserved structures were found in the promoters of three: Fur-family TF, WhiA-family TF and XRE-family TF (**Figure 6B**). A functional analysis of the respective operons showed that the Fur-family protein may regulate AP-endonuclease and respond to oxidative stress. Transcriptomics data are in accordance with this hypothesis. The WhiA-family protein may regulate thioredoxin-reductase and the SecG subunit of the protein export system. The XRE-family TF and the whole subsequent operon of restriction-methylation system is common in *M. gallisepticum* and *M. hominis* and was acquired by either species through horizontal transfer. Thus, the XRE-family TF is most likely a regulator of its own operon and may play a role in the global regulator controlling the methylation of the genomic DNA.

*Mycoplasma gallisepticum* and related mycoplasmas have unique genomic regions (gene clusters of membrane hemagglutinins of the VlhA family). The regulation of their expression is tightly controlled by repetitive GAA sequences upstream of the genes. The length of GAA was shown to be an important determinant of their expression level (Glew et al., 2000; Liu et al., 2002). Due to the high homology of 5′-UTRs of *vlhA* genes, reads from 5′-ERS libraries mapped to multiple loci, which impedes the correct measurement of a particular promoter activity. To measure their transcription level, we used transcriptomics data from our previous work (Mazin et al., 2014). Due to the high homology and mosaicism of *vlhA* genes, we used coverage by unique reads. Compared with the total coverage, a unique read resulted in less absolute values; however, tendencies were retained (**Supplementary Table S7**). VlhA genes can be classified into three groups on the basis of their expression level. There are two major, three medium and other minor *vlhA*. The transcription level between groups differs by at least one order of magnitude.

An analysis of *vlhA* promoter regions revealed conserved sequences including a deviant -10 box of a GCGAAAAT sequence (**Supplementary Figure S1**). This type of -10 box was identified in 29 genes including the major ones. Because this sequence is non-optimal for the sigma-70 but major *vlhA* transcription is extremely high, we propose that an alternative sigma factor may be involved in the expression of *vhlA* genes. We identified two additional conserved regions in addition to the GAA tract and -10 box. One is located upstream of the GAA tract and another in 5′-UTR. Disruption of the GAA tract is associated with the loss of other motifs and the reorganization of promoters from alternative to regular promoters. We identified that RNAs of medium-transcribed *vlhA* genes originate from promoters with a disrupted GAA tract and regular -10 boxes. We conclude that these genes left the control of the specific *vlhA*-regulating system.

One of the major *vlhA* has a 12-GAA tract, which is in accordance with previous data (Liu et al., 2002). However, another features a 14-GAA tract. Because there is a minor *vlhA* with the 14-GAA tract and conserved promoter, the GAA tract length cannot completely explain the regulation of *vlhA* transcription. Major *vlhA* genes are highly homologous (98%) and have one of the lowest levels of antisense transcription among *vlhA*. We propose that *vlhA* transcription is controlled by both GAA length (positive regulation) and antisense activity for their respective genes (negative regulation). GCW_01940 appears to be the primary major *vlhA*. The high transcription of GCW_01940 drains antisenses from highly homologous GCW_03350, which results in its derepression.

### Heat Stress Response Invokes the Differential Transcription of Multiple Regulons that Are Not Directly Involved in Heat Stress Defense

Previous observations demonstrated that particular stresses may result in the differential expression of numerous genes. This response may be stochastic or specifically controlled. After the reconstruction of the transcription control networks in the studied species, we applied a heat stress model to study the transcriptional response of the known system of regulators. Using quantitative 5′-ERS data, we measured the differential activity of promoters. We identified a total of 157 differentially expressed promoters in *A. laidlawii*, 30 in *S. melliferum*, and 195 in *M. gallisepticum*. Strikingly, in the well-annotated TF network of *A. laidlawii*, heat stress induced nine known regulons in addition to the regulon of heat-shock repressor HrcA. Two of these additional regulons included more than one operon. The induced regulons covered a broad range of functions including membrane transport, peptidases, and the metabolism of aminosugars and nucleic acids. However, the upregulation of these promoters was not comparable with the activation of HrcA-dependent promoters, which was approximately 100-fold. The maximum upregulation of other TF-controlled regulons was 10-fold (ABC-transporter controlled by ACL_RS02085). This observation raises the question of whether this response was non-specific but mediated by TFs.

We previously demonstrated that heat stress in *M. gallisepticum* leads to the differential expression of numerous genes. Although regulatory mechanisms of some of these were proposed, there remain numerous genes with unexplained upregulation. The example of *A. laidlawii* demonstrates that particular stress may impact numerous components of the regulatory network (likely non-specifically). A similar effect of multiple regulon induction in heat stress was observed for *M. gallisepticum*, which induced regulons of HrcA (twofold), MraZ (twofold), and GCW_02350 (fivefold).

Heat stress induces multiple promoters in addition to those directly responsible for the heat stress defense in all three species. Promoters controlled by the same TF behave coherently. However, there are multiple upregulated promoters lacking a predicted TF. The behavior of some of these promoters may be explained by a core-promoter structure, where heat stress favors to non-optimal promoters with a high activation barrier. However, others are likely controlled by yet unknown mechanisms. The amount of overall transcriptional noise during both logarithmic growth and heat stress increases with genome reduction. Generally, there is a great difference in the overall heat stress response in TF-rich *Acholeplasma* and reduced *Mycoplasma*. The first demonstrates substantially greater difference (orders of magnitude) between an adaptive and a noise-like response. Better transcriptional control is likely a result of the more pronounced promoter structure of *A. laidlawii*; in *M. gallisepticum*, any increased or reduced promoter-resembling sequence may initiate transcription.

Heat stress in *S. melliferum* produced different results. None of the identified regulons were induced. The most upregulated genes in *S. melliferum* represent the *groESL* operon (10-fold). However, a lack of CIRCE in the respective promoter indicates the presence of an alternative regulator. This observation is in accordance with the degeneration of multiple CIRCE in *S. melliferum*. Alternatively, the parameters of heat stress for *S. melliferum* could be not optimal for this particular experiment.

## Discussion

The combination of experimental whole-genome promoter identification and cross-species conservation analysis resulted in the identification of a substantial number (>50%) of the transcriptional control network components in the studied species of Mollicutes. Gene expression regulation can be divided into two types based on its aims: regulation in response to the external conditions and regulation of the housekeeping processes. A fundamental question remains regarding whether the cell’s functional core requires any regulation of gene expression or if its stability may be solely achieved through a perfect balance of promoter and RBS strength, codon usage and secondary structures within RNA as well as optimal protein-protein interactions within this core. Attempts to derive minimal gene content of the cell using a comparative genomics approach resulted in a set of genes that is too small to sustain the living cell (Koonin, 2003). Hence, there is a minimal set of functions rather than a minimal set of genes. In this work, we attempted to identify common functions that require the regulation of genes expression in a clade of bacteria that are a close approximation to a minimal cell. Most of the identified regulators in the studied species control responses to external conditions; thus, respective functions are not conserved between different clades of Mollicutes (inhabitants of different ecological niches with dissimilar environmental challenges).

However, there are two conserved functions. The first is protein stability maintenance. The respective regulator HrcA is the most conserved TF in all Mollicutes. The second is cell division control. This is an example of the phenomenon by which the same function is implemented using different sets of regulators. There are two cell division control regulators in Mollicutes: MraZ and, likely, WhiA. MraZ is conserved in spiroplasmas and mycoplasmas, whereas WhiA is conserved in all Mollicutes. The role of MraZ is more or less known (Fisunov et al., 2016) and appears to be widespread in Bacteria (Eraso et al., 2014); the function of WhiA as a cell division regulator was demonstrated only for *Streptomyces coelicolor* (Jakimowicz et al., 2006). In this bacterium, the WhiA-family TF was shown to regulate *parAB* and *ftsZ* genes. The WhiA homolog in Mollicutes does not reside within its hypothetical regulon. However, the identification of a conserved motif in the promoters of genes involved in DNA replication in acholeplasmas and spiroplasmas corroborates the hypothesis that they are controlled by a TF, and the most likely candidate is WhiA. The exact role of WhiA TF in Mollicutes has yet to be experimentally proven.

There is a TF conserved in most Mollicutes; however, its functions are obscure. The TF is a YebC/PmpR-family protein. It appears to be significantly involved in different functions in various organisms. Its putative binding site in *A. laidlawii* was identified. The TF controls an inorganic pyrophosphatase gene and genes with unknown functions. A cross-species gene conservation study demonstrated that the YebC transcription factor may regulate resolvase complex (RuvABC), most likely the RuvC subunit (Zhang et al., 2012). RuvAB is conserved across Mollicutes, but RuvC is functionally displaced by RecU. A cross-species conservation analysis of spiroplasmas and mycoplasmas demonstrates conserved motifs in the promoters of recU that are similar between these two clades but that differ from the clade of *A. laidlawii.* Another work showed that YebC/PmpR-family TF regulates quorum sensing in *Pseudomonas aeruginosa* (Liang et al., 2008). Thus, its homologs in different clades of Mollicutes may play distinct roles. An experimental approach of the artificial mycoplasma genome synthesis by Hutchison et al. (2016) corroborates our data on TFs conservation. TFs retained in a JCVI-syn3.0 minimal genome set include HrcA, WhiA, MraZ, and Fur homologs, whereas YebC/PmpR was proven to be dispensable.

All studied species have Hpr kinase, a component of the global regulation of carbon metabolism, but only *A. laidlawii* (and other Acholeplasmatales) has a homolog of the carbon catabolite repressor CcpA (ACL_RS05870). Its regulon consists of three operons involved in the transport and metabolism of carbohydrates. In contrast, spiroplasmas and mycoplasmas appear to lack a respective transcriptional control system. The function of CcpA in these species appears to be played by an unknown TF; alternatively, Hpr kinase may solely regulate carbohydrates transport.

In Mollicutes, regulons most frequently contain one operon (rarely more) (**Figure 6C**). *A. laidlawii* demonstrates a substantial number of multi-operon regulons; however, they are rare in other species. The overall reduction of Mollicutes likely resulted not solely in the reduction of the number of TFs but also in a decrease in the operons amount per regulon. However, the most obscure aspect of the studied networks includes isolated TFs. Their regulons are unknown and can be very broad. A reduction in the regulatory network in Mollicutes correlates with an increased amount of antisense and noise-like transcription. The number of noise transcripts increases with AT content, which increases the fraction of AT-rich promoter-like sequences across the genome [21] and with the decay of core-promoter determinants such as -35 and Ext elements. This phenomenon is also observed in *Helicobacter pylori*, another genome-reduced bacterium with genome-wide antisense transcription [8]. The structure of the *H. pylori* promoter is similar to that of *S. melliferum*, and the number of transcriptional regulators in genomes that comprises (17 and 22, respectively) is very similar. Noise-like transcription appears to impose a negative effect on fitness because it wastes resources; however, the degenerative evolution of Mollicutes led to its increase. In pathogenic bacteria, asRNA have diverse functions and often regulate their virulence and host interactions (Papenfort and Vogel, 2010). The comparison of non-coding transcriptomes of closely related pathogenic and non-pathogenic Listeria revealed the prevalence of species-specific regulatory RNAs in pathogenic bacteria (Wurtzel et al., 2012). Thus, we propose that antisense transcription may represent an important layer of transcriptional regulation that shows higher plasticity than the TF-based regulation. The emergence and decay of novel antisense promoters in an AT-rich genome may occur dynamically during the evolution and adaptation to the particular host via the acquisition of SNPs in promoter sequences.

From the functional point of view, the transcription control networks of the studied bacteria reflect their lifestyle. *A. laidlawii* is a saprophyte (initially isolated from sewage) and is rich in TFs controlling various transporters and membrane components. *S. melliferum* lives in two hosts: plants and insects, where various carbohydrates are the primary nutrients either in the phloem or hemolymph. Consequently, *S. melliferum* have a number of TFs controlling the transport and utilization of a broad range of carbohydrates and the specialized TF-controlling catabolism of pantothenate during persistence in the phloem. Similar to other mycoplasmas, *M. gallisepticum* lives in an animal host rich in all nutrients. Most TFs with identified binding sites in the three species are involved in metabolism control. The progressive loss of the conserved TFs from *A. laidlawii* to *S. melliferum* and *M. gallisepticum* is associated with a drastic loss of metabolic pathways. The simple metabolism of *M. gallisepticum* likely does not require any transcription-level regulation. Instead, at least two TFs in *M. gallisepticum* were predicted to control functions involved in the response to oxidative stress. The induction of reactive oxygen species is an important mechanism of antimicrobial response in the immune system; thus, it may be crucial to *M. gallisepticum* and other vertebrate pathogens in regulating respective defense systems. Additionally, *M. gallisepticum* developed a specialized system (Liu et al., 2002) including a yet unknown alternative sigma factor to control interaction with the host and immune response evasion. Stress response regulators are relatively underrepresented in the identified set of TFs. Apart from the ubiquitous chaperones repressor HrcA, only *M. gallisepticum* has specific regulators of oxidative damage. We hypothesize that response to various stresses in other species may be mediated by alternative sigma factors or global regulators such as SpxA (Kajfasz et al., 2015). Low TFs conservation in mycoplasmas and the existence of specialized transcription control systems led to proposing that a large number of regulators in reduced Mollicutes may remain undetected.

The identification of the regulatory network is based on two approaches. The first is based on the identification of genes coregulated in a panel of conditions and the subsequent identification of genetic determinants in their promoters. The second starts with the identification of binding sites of potential TFs. The first is relatively easier, especially for a small genome. However, a high-throughput analysis of stress panels of *M. pneumoniae* and *M. gallisepticum* did not result in the elucidation of novel TFs. We propose that the TF-based approach may partially resolve this situation. TFs of reduced Mollicutes tend to control single operons and even single genes. However, there are numerous genes with unknown functions in their genomes; some of these may represent TFs. Thus, the transcriptional control network in these bacteria may consist of broad range of TF with narrow regulons. The identification of MraZ TF provides grounds for the potential existence of novel TF families that are non-homologous to those currently known.

## Author Contributions

GF: Study design, conduction of the experiments, data interpretation, drafting of the manuscript. IG: Conduction of the experiments, data interpretation, drafting of the manuscript. DE: Conduction of the experiments, data interpretation, drafting of the manuscript. TS: Conduction of the experiments, data interpretation, drafting of the manuscript. AN: Conduction of the experiments, drafting of the manuscript. VG: Study design, data interpretation, drafting of the manuscript.

## Conflict of Interest Statement

The authors declare that the research was conducted in the absence of any commercial or financial relationships that could be construed as a potential conflict of interest.
