# Supplementary material for: Reconstruction of Transcription Control Networks in Mollicutes by High-Throughput Identification of Promoters
Source: Front Microbiol. 2016 Dec 6;7:1977. doi: 10.3389/fmicb.2016.01977 (PMC5138195; doi:10.3389/fmicb.2016.01977)
Supplement: Supplementary file 2 [file Data_Sheet_1.PDF]

Supplementary tables

Supplementary table 1 – Transcription start sites of *Acholeplasma laidlawii*

See file Supplementary tables, Table 1 - TSS A.laidlawii
Strand: Strand of the TSS
Position: Position of the first nucleotide of the RNA
Tag: Locus tag of the gene, which the TSS belongs to
Control1-2: TSS coverage under exponential growth (two replicates)
Heatshock1-2: TSS coverage under heat stress (two replicates)

Supplementary table 2 – Transcription start sites of *Spiroplasma melliferum*

See file Supplementary tables, Table 2 - TSS S.melliferum
Contig: Contig or plasmid ID of *S. melliferum KC-3*
Strand: Strand of the TSS
Position: Position of the first nucleotide of the RNA
Tag: Locus tag of the gene, which the TSS belongs to
Control1-2: TSS coverage under exponential growth (two replicates)
Heatshock1-2: TSS coverage under heat stress (two replicates)

Supplementary table 3 – Transcription start sites of *Mycoplasma gallisepticum*

See file Supplementary tables, Table 3 - TSS M.gallisepticum
Strand: Strand of the TSS
Position: Position of the first nucleotide of the RNA
Tag: Locus tag of the gene, which the TSS belongs to
Control1-2: TSS coverage under exponential growth (two replicates)
Heatshock1-2: TSS coverage under heat stress (two replicates)

Supplementary table 4 – Transcription factors of *Acholeplasma laidlawii*

See file Supplementary tables, Table 4 - TFs A.laidlawii

Supplementary table 5 – Transcription factors of *Spiroplasma melliferum*

See file Supplementary tables, Table 5 - TFs S.melliferum

Supplementary table 6 – Transcription factors of *Mycoplasma gallisepticum*

See file Supplementary tables, Table 6 - TFs M.gallisepticum

Supplementary table 7 – Transcriptional control of *vlhA* genes in *M. gallisepticum*

See file Supplementary tables, Table 7 – *vlhA* genes
Tag: Locus tag of the *vlhA* gene
sense coverage: coverage of the respective gene calculated based on all reads
antisense coverage: coverage of the respective gene calculated based on all reads in antisense direction relatively to the gene
GAA length: the length of GAA-tract upstream to the gene
sense unique coverage: coverage of the respective gene calculated based on unique reads only (mapped into no more than one location)
antisense unique coverage: antisense coverage calculated based on unique reads only
Promoter: sequence near respective TSS, -10-box centered (+50 to -44 relatively to the -10 box)

Supplementary materials

Supplementary materials 1 – promoters' alignments

Putative TF binding site is shown in green, promoter (-10 and -35 boxes) is shown in yellow, putative binding sites of other TFs if present are shown in grey.

WhiA(ACL_RS06640/SPM_004315), sequences in the *parA* promotor

```
A.brassicae       TTCCCACATTTTGTGTGGATAACTTTATCCCCCAGGAGAGACGCTTTTATGTTACTATT
A.equifetale      TATCCACAAATAATGTGGAAAACTTTTTAACATTTTTTGTATGTTGGTGTGCTATGATA
A.laidlawii       TATCCACAAATAATGTGGAAAACTTTTTAATAAATTTG-TCGTTTCTTATGCTATCATA
A.palmae          TATCCACAAATAATGTGGGAAACTTTTTAATAAATTTG-TCGTTTCTTATGCTATCATA
A.oculi           TATCCACAAAAAATGTGGGAAACTTTTGAAACTTTTTC-CACATTTATATGATATTCTA
                  *  *****     *****  ******                  *  *  * *    *
```

```
S.melliferum      AATGTGGAAAACTATTAATTTTTATTAATTTTACTCATTTTTTAGTACTTTTCCACA
S.kunkelii        AATATAGAAAACTATTAATTTTTATTAATTTTGCTCATTTTTTAATACTTTTCCACA
S.eriocheiris     CCTGTGGAAAACTTAAAAATTAAAGCGATGATTAAAAAAGACTATGACTTTTCCACA
S.syrphydicola    TTTGTGGAAAACCTAAATTTTTAGCAAATTAAAAATAACTTTTGCGACTTTTCCACA
S.chrysopicola    TTTGTGGAAAACCTAAATTTTTAGCAAATTAAAAATAACTTTTGCGACTTTTCCACA
                    * * ******    *  **      **             *    **********
```

```
S.melliferum      TCTTTTATTTATAATTAATAACGATAAAAACTATTGGAGATGAAATTA----
S.kunkelii        TCTTTTATTTATAATTAATAACGATAAAAACTATTGGAGATGAAATTA----
S.eriocheiris     TATTTAT-TTATAATAAATATTGGAATAAATAATTGGAGTGAATTAGAAACA
S.syrphydicola    TCTTTCT-TTATAATAAGAATTAGAAAAAAATGGAG---TGAAAGGCA---
S.chrysopicola    TCTTTCT-TTATAATAAGAATTAGAAAAAAATGGAG---TGAAAGGCA---
                  * ***   ******* *  *     * ***            *
```

ACL_RS01290

```
A.hippikon        TAAAACGTTTACATTCATGTTATAATAATTTAAAGAATAAGAA
A.equifetale      TGAAACGCTTTCAACCGTGATATAATAAGTGCATGAGTAAAGA
A.laidlawii       AAAAACGGTTTCACTCATGTTATAATATAGATGAGGAAGATAA
A.oculi           AAAACCGTTTACACTCATGATATAATATAGAAGAAATAGATAA
                    ** ** ** **  * ** *******            *  *
```

```
ACL_RS06070

A.granularum     TTTATTACGGGTTCTATTAATTGACAAACCACCTAACAGTGGTTACAATTAAACTATAAC
A.laidlawii      TTTTTGCTGGTCTATATTAATTGACAATAAACCTAACAGTGGTTACAATAA---------
                 *** *   **  * *************   ******************* *

ACL_RS05980

A.hippikon       TAGTTTATATAAATATAAAGAAAGTTTAGTATGAATGCATTTTCATCAATTATCATTAGA
A.laidlawii      ----------------AAAGTAAGTTTAGAATGAATACACTTTCAATTAATAAGTTATTG
A.oculi          ---------GAATTATAAAAAAGTTTAGAGTGAATACACTTTCACTTCAATAGTTATTG
                                 ***  ********  ***** ** *****          *

A.hippikon       TATTGTTCATCATACTCATGTTATAATAATAGATGAAAAAACGATTA-------------
A.laidlawii      GCAACAATTTTATGTTCATGGTATAATATGACATGGAAAAACGTTTAACATAAGGAGAAA
A.oculi          GAAACGTTTATAAAAACATGGTATAATATCATTTGGAAAAACGATAATATAAAAGG----
                            *    **** *******  *  ** ******* * *
ACL_RS06425

A.oculi          TCACTTTACATTAAATTTACATACACCTTACTTTAAATGGTTATTTTAAAGATTTTAAAT
A.laidlawii      TAACTTTACAAAATCTTTACAATCTCCTTACATCCTTTGGTTTTTCTACCGATTTTAAAA
A.brassicae      GTTTTTTACAAGACCTTTACAATAACTTTTCAAAGGATGGTATTTTGAACATCTTTTTTT
                     ******     ******    * ** *      ****  **  *     ***

ACL_RS03855

A.brassicae      -GTATAATGAAAGTGACAACAGTGTCACAAAAGGA--
A.oculi          TATAATATAAATATGACAACCTTGTCATTTTGAGGGA
A.laidlawii      TATTCTATAAATGTGACAACCTTGTCATAACAGGAGG
                   *   ** **  *******  *****      *

ACL_RS02515

A.laidlawii      TATATGAATAGAATGTAAAT-AGTGTATAATGAATCTATGTTCACATTTCAGTGAAATCA
A.oculi          CGGATGTGTATATTGTCAATCCTTCTTTGATGAATATATATTCATAAACCACATACCACC
                    ***  ** * *** ***   * * * ****** *** **** *   **   *   *
```

```
ACL_RS02085

A.laidlawii    GAGTTTACAAATCA------TTTTTTATGGCCTATAATAGTTAGGTACCTAACTAATAAG
A.modicum      GTATTTTTATTTGCTTTTTTAAAATAATAGTCTATAATAGTTAGGAGCCTAACAAT----
A.granularum   ATGTTTACAAATGCTTA-------AAAATACGTATAATAGTTAGGTACCTAACTAATTAA
                  ***  *  *              *     *************  ****** *

ACL_RS03205

A.granularum   TAATTG-TAAATTGATTGATAAAAGTAATATAATTATAGTATATAGTATAAACTATTT--
A.laidlawii    TAATTGTTTGACTTTTAGTAAATTGGACTATAATGATAGTATACACTATAAACTAATGGT
               ****** *  * *  * *  **  * * ****** ******** * ********* *

ACL_RS06135

A.hippikon     TAAGTGCTAAATTATTGACTATTATAAAAAAATCAATATAATTGTTAGCATGCTAACTAA
A.granularum   TATAGGCAAATTTATTGACAATATTTTTTTAAATATATAATCATTAGCATGCTAACTAT
A.laidlawii    CAAAAACAAATGATTTGACATTATCTATAAAACTATATAATCATTAGCATGCTAACTAT
                         *    * **    *****  * *   *******  ***************
ACL_RS00415

A.granularum   TTAATATATGTTA----AAATACTATAGATATATTTTGAATATCAAAGTAT---------
A.oculi        AAATAATGTGTTACACTTAATTTAC-AAATATATTTTGATAATCAAAGTAATTTTAA---
A.laidlawii    TAATTATGTGATACACTTAATCTATAAAATATATTTTGATACTCAAAGTATTTTAAGGGA
                 *  ** ** **     ***     * ***********   ********

ACL_RS02835

A.oculi        CATATTTTGCGCATAAAATGCATATATGTGGAAATTAATTCGAATTCGAAATATAATGTA
A.laidlawii    CCACAATTTGCCTAAAAATGCATATTGATGGAAATTATTTCGAATTCGAAATATAATGTA
A.granularum   CCACAATTTACCAAAAAAAGCATATTCATGGAAATTATTTCGGATTCGAAATATAATGTA
               *     **   *  **** ******   ********* **** *****************

ACL_RS05200

A.laidlawii    AATAATTCATACAAATAGTATACAATTAAATTATTCAATAAACGAACAATAAGGGGAGAT
A.granularum   TAAAATTATAATAAATAGTATAAAATATGATTATTCATTAAACGAACAATAA--------
A.brassicae    TAATAATCAAATCTTTAAGATAAAATGTAATCGTTAATTAAACGAACAATG---------
                *  * *   *    **  *** ***   **  ** * ************
```

```
ACL_RS01910

A.oculi         -------CAATAAAGATAATCATCAATATATAAATGATTATCTTTT-ATTGTATAATGGT
A.laidlawii     AAAATACTTTTAAAGATAATCATTCTTGTTATAATGATTATCTTTT-AATGTATAATAGA
A.granularum    ----ATAAGTTAAAGATAATCATTGATATTTAAATGATTATCTTTTTCATGTATAATAGA
                          *************   * *   ********** * **  ******** *

ACL_RS05425

A.laidlawii     TTTTGCGAATCATTTGCAAAAGTATTATATCACACAAAATAGACTTTGCAAACGATTTGCAAA
A.granularum    TTTTGCAAATCGTTTGCAAAGTCTATTTTGTGTGATATAATACTTTTGCAAATGATTCGCAAA
A.oculi         TTTTGCAAATGATTTGCAAACTGTATTTTTCATGATATAATAATTTTGCAAATAATTCGCAAA
                ****** ***  ********     * *          * *     ********  *** **

ACL_RS02790

A.oculi         ACAATAAATATATATTTATTATTGTGTGATTTTTTGTATAAATAGTGGTATAATTTTTATAA---
A.laidlawii     ATTTAGTTTTTTGTTCACATAATGTGTGATTATTTATATAAATAATGGTATAATTTTTATAATTA
A.granularum    AAATAATTTTAATTAATTATATTATGTGATTTATTATATAAATAATGGTATAATTTTTTATAATT
                *       *          ** * *******  ** ******** *************   *
ACL_RS03695

A.laidlawii     ------AATTTGCTTACGAATACTTTAATAAAGCATGG----------------------
A.granularum    TATTTTAACATGT-TATAGATACTTTTATAAAGTATTTGCGTAACTTTATTGTTAAATTT
A.brassicae     CATCA-------A-AAAAGGGTATTTTATAAACCAACAAGGACAT-CTGTTC----TTTT
                               *        *** *****   *

ACL_RS03555

A.laidlawii     -------ATTTTTGAGAAAACGATTTAGTAAAATACGAAAACGTTGTAAAATGATGAATA
A.oculi         TGAGCATTTTTATAGGAAAACGCTTTATCTATTTACGAAAACGTTGTAAAAAAGTTTATA
                        *** *  ******* ****    *  *****************   *  ***

A.laidlawii     CTATAGTTGTAATCGTTTTCATAAGGTGATATAATAAAAATGTCTATTAATTATACATTC
A.oculi         TTTTTGTTGAAATCGTTTTCACATAGTGTTATAATAAAACTGAGATATAAATCGAAATCC
                 * * **** *********** *  *** ********** **      *** *  * ** *
```

ACL_RS00185

```
A.brassicae     TTAAGATGATTACGTAAACGATTTCGTTTTAAGATTGT
A.oculi         AGAATATAGTTGCGCAAACGTTTAAGTAAAAAATAAGA
A.laidlawii     AAAATATAGTTGCGCAAACGTTTACGTCGATA------
                  ** **  ** ** ***** **  **    *
```

ACL_RS05870

```
A.hippikon      TTTATGAAAAAGTTTTCATAATGTTTCATAATTTGTTGTAAGCACTTCCATCTTATGTTATAATT
A.granularum    ATTATGAAAATTTTTTCATTTTGTTACATTTAGTATTGTAAGCACTTCCATTTTATGATATAATT
A.oculi         TTTTATGAAAATTTTTCATTTTGTTTCATAACATATTGTAAGCACTTCCATTTTATGATATAATC
A.laidlawii     TTTATGAAAGTTTTTACATTTTGTTACATTACATATTGTAAGTGCTTACAATATATGATATAATT
                 **    **   *** ***  **** ***    * *******  *** ** * **** ******
```

ACL_RS02340

```
A.granularum    TAGTATTGTGTAATACACGGTACATGTGGTATTATAGTAGTGTGTATTACACAATAC-
A.laidlawii     ATGTATTGTGTTTTACACAGTACATGTGATATTATAGTAGTGTGTTAAACACAATACA
A.oculi         AGGTATTGTGTTTAACACAGTACCTATGATATACTAGTTATGTGCAATACACAGTACC
                   *********   **** **** * *****   ****  ****    ***** **
```

ACL_RS02740

```
A.brassicae     TTTAACACACAACATTTTCTTATTGACAATATTGTATGTCATACACTATAATAA------
A.oculi         TTATTTTTTTAATGTTTTTTATTTGACAATATTGTATGTCATACAGTATAATAAAAAT--
A.laidlawii     TGTATTTTATAATGTTATTTATTTGACAATAGTGTATGTCATACAGTATAATTAAAAATG
                *         **  ** * *  ********* ************* ****** *
```

ACL_RS06835

```
A.hippikon      ----CCTTAAGAAAGTTTGAAGTTTATCTTTGACTCACTATACTATGCAATATATAATAT
A.laidlawii     TAGGTTTTTATTTTTATTTATTATATTACTTGACACATAATACTATGCGACATATAATAT
A.granularum    TTTTGTTTAGATATATTTAACTATATTACTTGACACATAATACTATGCGCCGTATAATAT
                      **        ** *   *  *  ***** **  *********    ********
```

ACL_RS03765

```
A.laidlawii     AGTTGGTGTCGTAAAAGATTATATAGATTAATATAGAATGA----ATCAAGTTTATAATT
A.granularum    --AAAATATTTTAAGAAAACACATTGACAGCTATCGATATGAGATATATAATTAACATAT
A.modicum       ATTTGTAGATATAAATTACTATATTGACAGATATCAATATCAACTGTATAATTAATACAT
                           ***   *  * ** **    ***  *         *  * ** * *  *
```

ACL_RS03730

```
A.laidlawii      ----------------TTACAAAATCGTTGTATAATAGAGACAACGCTTACAGTGATAAG
A.oculi          TAAATCTATAAACAAATTAAAATTTAGTTGTATAATAAAAACAACGTTATAAAAAGAGG-
                                 *** **  * *********** * ****** *   *
```

ACL_RS06235

```
A.hippikon     GTTGTTTTTATTGACTCCGCA------GTTTATTGTGATACAATATATCTATATGAATGAAC
A.laidlawii    CATATAACTATTGACACAAGAGAAAAACAATAATATAATAAATATACAACGTATGAATGCTT
A.oculi        CATATAACTATTGACACAAGAGAAAAACAATAATATAATAAATATACAACGTATGAATGCTT
A.brassicae    CATATAACTATTGACACAAGAGAAAAACAATAATATAATAAATATACAACGTATGAATGCTT
                 * *   ****    *   *         ** * * *** **        ********
```

ACL_RS07175

```
A.laidlawii     GTGGTAAAATAC--ATCTAAAGTGTTCTACTGTAAGTTATTTATAAATTCATGTAACTTATAAG
A.granularum    TTGGTAAAATGAGGCTATAAAAAACATTGCTGTAAGTTATTGCTCATAACATGTAAGTTATTTG
                 *********     * ****      * ************  *     ******* ****  *

A.laidlawii     ATAAACCTTGCTACTTTAAACTAGGTTTTTGTAAAATCTAGA
A.granularum    AATTTACTTTATTAAAAAA--ATATTTATATTAAACTCTAGA
                *     ***  *     **      ** *  ***********
```

ACL_RS04235

```
A.laidlawii     ----------ATTGTGACACGACATATATGTCGTATAGTGAAAGGAGTAA
A.oculi         ATAACATTAAAAATGAACACGACATATATGTCGTATAATGATCGGG----
A.granularum    ATATCAAAACAAAACAACACGACATATGTGTCGTATAATGAA--------
                          *     *********** ********* ***
```

ACL_RS05305

```
A.laidlawii                      CTATTTATATTGACTTAATATGTATAATCAATTATAATGTAA
Lachnospiraceae bacterium        CAAAAAAAATTGACCTATCAGGTTAATTATGATACGA-----
Olsenella sp. oral taxon 807     TCTCAAGAATTGACTTATGAGGTCAATCATGTTATCC-----
                                         ****** **  * **  *      **
```

ACL_RS04610

```
A.laidlawii             GGAGATAATATCGCAAGTTCTTGCGAATAAACATTAATTTCGTAT
Blautia hansenii        TGGACATAATTCGCAAGAACTTGCGAATTATGTCAAAATATATTG
Eubacterium siraeum     ACCGGAAAATTCGCAAGAAGTTGCGAATTTTATCTATAAATTTGC
                               *  *******   ********       *      *
```

ACL_RS03655

```
A.oculi        TAAAAATAGAGTTATTTTTGATTAATCTACTATGAGTAGAGTCGT-A-TTTGTGATAGAATAA
A.laidlawii    ACACAATAGAGTTAATTTTCAAGATTCTACTGGGAGTAGAATGAT-ACTTTATGATACAAT--
A.granularum   ACACAATAGAGTAAAAATTGATGATTCTACCCGTAGTAGAATCATTAATTTATGATAATATAT
                 * ******** *   ** *  * *****    ****** *  * * *** *****  **
```

ACL_RS05490

```
A.laidlawii                      GTTATAAATAATTGATAGTTGTCAATTTATTGATATAATGTAAT
Carnobacterium pleistocenium     TTGGGATATACTTGATTTAAATCAAGTACAATTAGTT-------
Carnobacterium inhibens          GGTGATTGTACTTGATTTAAATCAAGTCTATCAAAAA-------
                                         ** *****     **** *
```

ACL_RS06565

```
A.oculi         TATTCACTTTATACTTTACTACACTAAAGCAATAAAGTATAATTGGA
A.laidlawii     ATTACACTTTATACTTTACTACGGTAGAGTAATAAAGTATAATTAGT
A.granularum    CATATACTTGATACTTTACTACACTAGAGTATTAAAGTATAATTGAT
                  *  **** ************  ** ** * ***********
```

ACL_RS06770

```
A.multilocale   GGTATACTTTGTAAAGTAAGCACTTAAAAGTGCTATACTTA
A.laidlawii     AGATTACTATGTATTGATA---AATGTGCGTGATATAATCA
A.granularum    AAATTACGGCGTATTAATATGTTATGAATATGTTATAATCT
                    ***   ***     *     *     ** **** *
```

ACL_RS00230

```
A.oculi         ATGGGTAATAGAGATAGAAAAATCATAAAAATAGTGTTACAATAAGTTTGCTACGAGATA
A.laidlawii     ------GCAAAAGATAGAAAAATACCTAATTTAGTGATACAATGTATATGCTAAT-----
A.granularum    ATATATAAATAAAATAGAAAAAAGAAGTAAATAGTGATACAATGTATTTGCTAATAGAGG
                           * *********      *  ***** ******   * *****
```

SPM_001160

```
S.eriocheiris   TGTAAATTTTACCGACTAATGTAAATTTTATGTAAATTTTATAGTAAACTCTTATATT-------
S.melliferum    TGTAATTTTTTTATTAAAATGCAAATTTTTTGAAAATTTAATGCATTATAATTTTTAATAAAGGA
S.citri         ----ATTTTTTTACTGAAATGCAAATTTTTTGAAAATTTAATACATTATAATTTTTAATAAA---
                    * ****       **** ******* ** ****** **     *   ** *
```

SPM_001085

```
S.eriocheiris    TATAAAAGTTTAATGTAAAATTATTACAGGGATTCAGCAAATTATTTTCACAAT
S.poulsonii      TCTAATATGTTGTAAAATAGAAATAGTTGATATTATGAAAATAATTTTCATGGC
S.melliferum     CATAATATGTTGTAAAATAGAATTAGTTGATATTATGAAAATAATTTTCATGGT
S.citri          CATAATATGTTGTAAAATAGAATTAGTTGATATTATGAAAATAATTTTCATGGT
                   *** *  **     * *    *    *  ***  * **** *******
```

SPM_001110

```
S.poulsonii     ---GTTTTATGTTATTTTTGGTTGTTTTTGGTTACAAAGGATTTATAATAAGGATGGTGA
S.citri         TT-----------ATTTTTGGTT-TTTTTGGTTAAAAATAAGATATAATTACAGT-----
S.melliferum    GTTATTTTATGTTATTTTTGGTTGTTTTTGGTTAAAAATAAGATATAATTACAATT----
                             ****** * * ********** ***  *  ****** *   *
```

SPM_004435

```
S.eriocheiris   ATTATTGATATTTTTACATTTTTCCCTATAATTAACTTAAATGAAAATTAAAAAACAAAAAGTTTAGTATAACTAAACAAG
S.kunkelii      ACTATTGATATTTTTTTTGTTTTTTTTATAATTAAT-GTTGCCATAATTTAAAAA----AAGTTTAGTATAACTAAACAAA
S.melliferum    ACTATTGATATTTTTTTTGTTTTTTTTATAATTAAT-GTGGTCGTAATTTAAAAACAAAAAGTTTAGTATAACTAAACAAA
S.poulsonii     ----TTGATATTTTTTTTGTTTTCCCTATAATTAAT-GTGGTTGTAATTTTAAAACAAAAAGTTTAGTATAACTAAACACA
                    ***********    ****   *********          ****  ****    ********************
```

SPM_003420

```
S.eriocheiris   TTGATAATATCTATGTTATGATTTTAGTAAAT
S.melliferum    TTAATTTATTCTATGTTAAGATAAAAAAGATA
                ** **    *************   *
```

```
SPM_003510

S.poulsonii     GGAACATCGATATATTAATATTTTAATATATCGTTTTTTATTTGTATAATTAATAA
S.citri         TAAAAATCGATATATTA--ACATAAATATATCGATTTTAATTATATAATTATAAA
S.melliferum    TAAAAATCGATATATTA--ACATAAATATATCGATTTTATTTATATAATTATAAA
S.kunkelii      TAAAAATCGATATATTT--ATGTTAATATATCGATTTTAATTATATAATTATAAC
                  ** ***********   *  * ********* ****** ** ********  *

SPM_005475

S.kunkelii     --ATATTTTAAAAAATGTGTATTATAATCATGATATGGAAATGATTCCATACAGGAG---
S.melliferum   ------TTTAAAAAATGTGTATTATAATCAAGATATGGAAATGATTCCATACAGGAG---
S.citri        TTATATTTTAAAAAATGTGTATTATAATCAAGATATGGAAATGATTCCATACAGGAGATA
                     ************************ **************************

SPM_001155

S.melliferum       GAATAAATTTATTTGAT--ACAATGCTTATATAAAAAACTATTTAACTATTAAATTAACAAGATAATTATAGAAG
S.poulsonii        AAGTATTTTTTATTGATTTCCAAT------------------------TTAAAATTAATATTATGAGAAGTAAAG
                    * **  ***  *****   ****                         * ******* *  ** *  *   ***

SPM_000505

S.poulsonii      AGAAAATAAGGTATAGTTAAAAAAGAAGATATTATTTTAAATAAAATAATATAAGTTATC
S.melliferum     TAAAAATAGCGTATAGTTAATAG-AAAGAACTTATTTTAAATAAGATGTTTTCTTTT---
                   ******  ********** *   ****  ************* **  * *   **

GCW_02090

M.gallisepticum   AATAATTAAAATAAAACCAAGCAATAAAACCGCAATTGGTTTTATTTTTAT---------
M.imitans         AATAATTAAAATAAAGCCAAGCAGTAAAACCGCATTTGGTTTTATTTTTATCTATATATG
                  *************** ******* ********** ****************

GCW_02350

M.hominis         AAAGTGATAGTTTTGTGATATAGTTAAG
M.gallisepticum   TTTGTGTTAAAATAGTGTTCGATTTTGA
                     *** **   * *** *    **
```

Supplementary figures

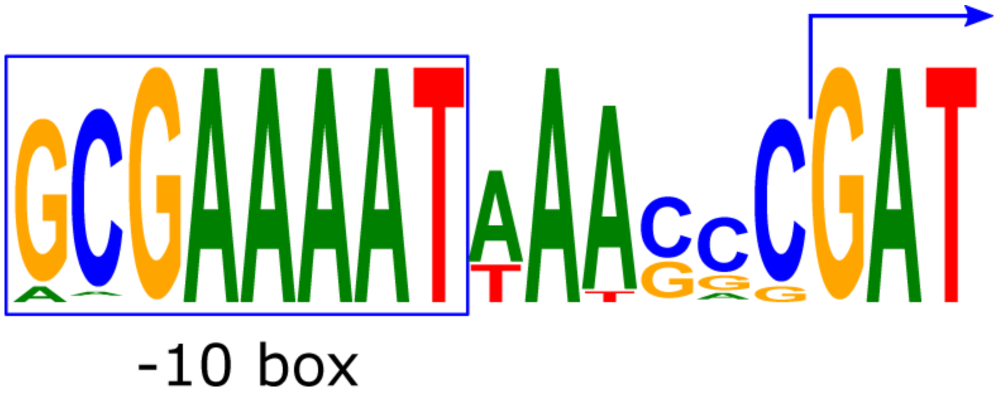

Supplementary figure 1. Structure of *vlhA* genes promotor. Arrow indicates TSS position, -10-box shows alternative sequence instead of consensus TATAAT.
